# Effects of Ai-Chi Practice on Balance and Left Cerebellar Activation during High Working Memory Load Task in Older People: A Controlled Pilot Trial

**DOI:** 10.3390/ijerph182312756

**Published:** 2021-12-03

**Authors:** Michal Nissim, Abigail Livny, Caroline Barmatz, Galia Tsarfaty, Yitshal Berner, Yaron Sacher, Raffaella Bodini, Navah Z. Ratzon

**Affiliations:** 1 Sackler Faculty of Medicine, School of Health Professions, Tel Aviv University, Tel-Aviv P.O. Box 39040, Israel; caroline.barmatz@gmail.com (C.B.); Galia.Tsarfaty@sheba.health.gov.il (G.T.); ynberner@post.tau.ac.il (Y.B.); ysacher@clalit.org.il (Y.S.); navah@tauex.tau.ac.il (N.Z.R.); 2 The David Yellin Academic College of Education, Teachers for Students with Complex and Multiple Disabilities, Jerusalem P.O. Box 96342, Israel; 3 Sheba Medical Center, Ramat Gan P.O. Box 52621, Israel; abigail.livnyezer@gmail.com (A.L.); raffaella.bodini@gmail.com (R.B.); 4 Sackler Faculty of Medicine, Department of Imaging, Tel Aviv University, Tel-Aviv P.O. Box 39040, Israel; 5 Sagol School of Neuroscience, Tel-Aviv University, Tel-Aviv P.O. Box 39040, Israel

**Keywords:** ageing population, aquatic therapy, balance, cerebellum, f-MRI, N-back

## Abstract

Background: Normal aging is associated with balance and working memory decline. From a neurobiological standpoint, changes in cerebellar functional plasticity may mediate the decline in balance and working memory for older adults. Mounting evidence suggests that physical activity is beneficial for decreasing aging effects. Previous studies have focused on land-based physical activity and research concerning the aquatic environment is scarce. This study investigated the effectiveness of Ai-Chi on balance abilities and cerebral activation during a high working memory load task among community-dwelling older people. Methods: A total of 19 people aged 65–86 years were allocated to receive Ai-Chi practice (*n* = 6), structured on-land Ai-Chi practice (*n* = 7) or guided-imagery of Ai-Chi practice (*n* = 6) for a bi-weekly, 30-min exercise session for 12 weeks. Balance was measured by the Tinetti balance sub-test and working memory was measured by the N-back test during functional-MRI scan. Results: The Ai-Chi practice group presented a significant change in balance between pre and post intervention (balance *t* = −4.8, *p* < 0.01). In the whole-brain analysis, during high working memory load task, the Ai-Chi practice group presented a decrease in left cerebellar activation. Region of interest analyses yielded similar results by which pre-cerebellar activation was higher than post-intervention (*t* = 2.77, *p* < 0.05). Conclusions: Ai-Chi is an available, non-invasive intervention method that may serve as a tool to improve cerebellar activation that in turn might improve balance. In addition, our findings may provide new insights into the neuronal mechanisms that underlie both motor and cognitive abilities.

## 1. Introduction

Normal aging is associated with a decline in both balance [1] and working memory abilities [2]. From a neurobiological standpoint, changes in cerebellar functional plasticity may mediate the decline in balance and working memory for older adults. Studies have shown an age-related decrease in the structural morphology and function of the cerebellum [3]. According to the traditional view of the functional organization, the cerebellum is mainly involved in the coordination of motor movements, maintenance of balance, and motor learning [4]. Recent studies have found that the cerebellum also plays an important role in higher non-motor cognitive functions such as working memory [5,6].

Various forms of physical interventions have been found to be beneficial to promoting balance and working memory, improving brain structure and function for older adults [7,8]. Tai-chi has been practiced as a physical exercise, mainly by older adults, because of its low speed. Tai-Chi has been used as an effective way of improving balance and reducing falls [9,10] as well as improving cognitive abilities such as memory in older adults [11,12]. These studies are promising but inconclusive.

The environment in which a physical intervention occurs might influence the results of the intervention [13]. Therefore, changing the environment to aquatic might result in changes in cerebellar activity, which might influence balance and working memory. The physical forces in the aquatic environment (for instance: specific gravity, the meta-centric effect, and thermodynamic) affect the immersed body in a different way than on land [14]. Immersion induced activation in motor and somato-sensory cortical areas [15] may enhance motor learning during the acquisition of motor skills. A magnetoencephalography (MEG) study of adults has shown improved verbal working memory following 28 days of daily aquatic physical practice. A significant interaction between time (Time 1 and Time 2) and group (on-land motor intervention, aquatic motor intervention, and non-motor intervention) was observed [F(2, 21) = 4.56, *p* < 0.05], demonstrating significant improved performance following intervention only for the aquatic motor intervention group (t(7) = − 4.96, *p* < 0.01). The improvement in verbal working memory was correlated with increased right cerebellar alpha power (r = 0.39, *p* < 0.05, *n* = 24). Greater cerebellar alpha power was correlated with improved performance in the digit span task [16]. 

In the present study, we propose an intervention program using the Ai-Chi method. Ai-chi was developed by combining techniques of Tai-Chi and Qi Gong and performing them in the water. Ai-chi is performed in shoulder-depth water and contains deep breathing and slow movements of the arms, legs, and torso. The method follows a sequence of 19 movements and progresses from simple breathing to coordinating movements of the arms, trunk, and legs [17]. In a previous study which sought to analyze the effects of an Ai-Chi program on fall-risk and verbal working memory in older adults with intellectual disability, results attest to the positive effects of both Ai-Chi and Tai-Chi on the fall-risk score, with the fall-risk score of the Ai-Chi group improving faster than that of the Tai-Chi group. Study findings also support the positive effects of Ai-Chi on working memory [18] and balance [19]. However, the neuronal mechanism underline of these changes was not examined. Adding to this body of research, we recently showed in a randomized control trial that participation in 12-week Ai-Chi practice was associated with improved balance and working memory abilities of community-dwelling older individuals. The differences in Tinetti balance (F (2, 39) = 10.03, *p* < 0.01), fall risk (F (2, 39) = 5.62, *p* > 0.05), digit span forward (F (2, 39) = 8.85, *p* < 0.01) and Corsi blocks forward (F (2, 39) = 3.54, *p* < 0.05) and backward (F (2, 39) = 6.50, *p* < 0.05) scores after 12 weeks between the groups were significant. The aquatic motor intervention group showed improved scores [20].

Based on the above, the first objective of this study is to examine the effects of Ai-Chi intervention for older adults on balance compared to identical intervention on-land and identical cognitive non-physical intervention. The second objective is to examine the effects of Ai-Chi intervention for older adults on cerebral activation during working memory tasks, compared to identical interventions on-land and cognitive non-physical intervention, in preparation for a fully powered trial. This enables the exploration of the neuronal mechanism that may mediate between balance and working memory.

## 2. Materials and Methods

### 2.1. Study Design

This three-arm pilot study is part of a larger study, which aimed to investigate effects of Ai-Chi on fall-risk, and hazard perception as pedestrians [20]. The current pilot study focused on the effects of Ai-Chi intervention on balance and cerebral activation during a high load working memory task. All members of the professional team involved in evaluating participants were blind to the intervention group to which participants were allocated.

### 2.2. Participants

**Figure 1:**
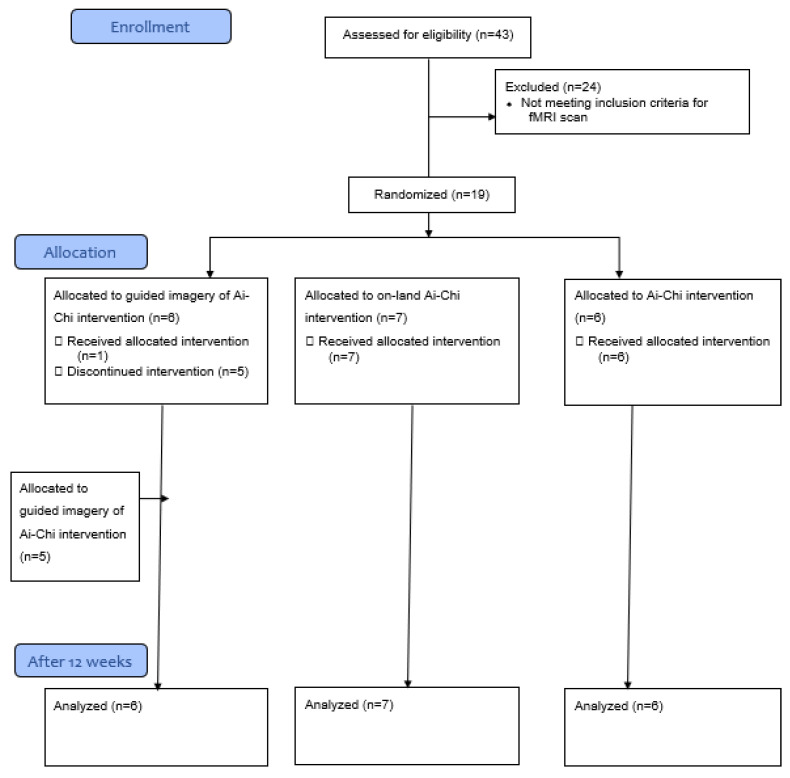
The CONSORT 2010 flow diagram.

A total of 19 older adults aged 65-86 years (M = 74.7 ± 6.58) participated in this pilot study (Figure 1). The inclusion criteria of this study were: aged over 65 years old, scoring below 10 on the Geriatric Depression Scale [21], and scoring above 24 in the Mini-Mental State Examination [22]. The exclusion criteria of this study were: a medical history of neurological, orthopedic, or psychiatric conditions causing permanent impairments, the use of drugs that may cause dizziness (according to manufacturer guidelines), absence of intervention exceeding two weeks, people who were claustrophobic, had metal in their body, and those who had a brain tumor.

The sample size was estimated for the larger study [20] based on digit span forward test, with 80% power and a standard deviation of 0.63, which can be achieved with a minimum of *n* = 10 participants in a group. However, locating participants over the age of 65 without metals (permanent makeup, pacemaker, etc.) was found to be a difficult task. Given the three year limitation on the study, we decided to discontinue the scans.

### 2.3. Outcomes

The following were the outcome measures utilised for this study. 

#### 2.3.1. Balance

The Tinetti balance, gait and fall-risk test [23] is a standardized evaluation of balance and mobility designed to determine risk of falls among older adults and has excellent inter-rater reliability and sensitivity. In this study, we focused on the balance score. 

Balance testing includes sitting balance, arising from chair, immediate standing, standing balance, balance with eyes closed, turning balance, neck turning, back extension, one-leg standing, push test, reaching up, bending down, and sitting down.

#### 2.3.2. Structural and Functional MRI (f-MRI)

f-MRI was conducted at the Alfredo Federico Strauss Center for Computational Neuroimaging on the Siemens Magnetom Prisma 3T Scanner with a 64-channel Head Coil, using the following measures:

Structural Imaging protocol: T1w images were acquired magnetization-prepared rapid acquisition gradient echo (MPRAGE) sequence with TR/TE/TI of 1750/2.59/900 ms, matrix os 224 × 224 × 176 mm, flip angle of 8° with isotropic resolution of 1mm3, covering the entire brain.

f-MRI imaging protocol: Task functional T2*-weighted images (BOLD contrast) acquired using a multiband gradient-echo echo-planar imaging (EPI) pulse sequence (TR/TE/flip angle = 3000/30/90° with FOV = 224 × 224 × 72 mm, and an acquisition matrix dimension of 112 × 112); 72 contiguous axial slices with 2.0 mm thickness were acquired, covering the entire brain.

The n-back working memory f-MRI task was presented to participants using the E-Prime 2.0 software (Psychology Software Tools, Inc., Sharpsburg, PA, USA). Stimuli were randomly presented. Visual stimuli was back-projected on a 32’ MRI-compatible screen and viewed through a mirror device. Responses were recorded with a four-batten response box from Current Design. 

The n-back procedure is a widely used f-MRI task in studies of working memory in healthy individuals [24]. In this study, we used the 22 letters of the Hebrew alphabet as stimuli, in two different memory load conditions (1-back, 2-back). The participants were asked to indicate whether a target stimulus matches a stimulus presented *n* (0, 1, or 2) stimuli earlier. In the 0-back condition, participants were instructed to press the button on the response box when the presented stimulus is a pre-specified target letter. The 1-back and 2-back conditions alternated with a 0-back condition to control for basic attention and sensory stimulation. There were twelve blocks consisting of three 1-back blocks, three 2-back blocks, and six 0-back blocks, with a 6-s blank screen interval between blocks. Target stimuli were presented for 33% of trials in each condition. Stimuli presented in 20-s blocks, during which each of 10 letters presented for 0.5 s, followed by a 1.5-s blank screen inter-stimulus interval. Before scanning, participants were trained in similar tasks presented on a desktop computer outside the scanner, to ensure that participants could perform above chance.

### 2.4. Interventions

The intervention protocol took place from 2018 until 2019 at Tel Aviv University, Sheba medical center and Raich Center, and included a 30-min exercise session conducted twice a week for 12 weeks, for a total of 24 sessions. Four instructors conducted the intervention. All instructors were certified hydrotherapist Ai-Chi instructors or Tai-Chi instructors and were trained for the intervention protocol to ensure identical intervention in all groups. In addition, a research coordinator followed the research protocol once a week.

#### 2.4.1. Intervention Groups

##### Ai-Chi

The Ai-Chi method, based on Qigong and Tai-Chi movements was selected [17]. For the present study, 16 movements were used from the Ai-Chi method. Illustration of the 16 movements can be seen in Supplementary Table S1. The first six movements were more static and symmetrical while the other movements were focused on continuously changing the center of gravity and center of buoyancy. The Ai-Chi intervention was conducted in a hydrotherapy pool (34 °C) approved by the Ministry of Health.

##### On-Land Ai-Chi

For the controlled comparison of the structured on-land motor intervention, 16 identical movements were used in the Ai-Chi method.

##### Guided Imagery of Ai-Chi

Participants in the guided imagery of Ai-Chi group practiced guided imagery of the 16 identical movements used in the Ai-Chi method (listening to the instructor’s voice) while sitting on a chair.

Both the on-land Ai-Chi and the guided imagery of Ai-Chi were conducted in a quiet room.

The guided imagery of Ai-Chi group was chosen in order to control for the motor aspects of the intervention, while the on-land Ai-Chi group was chosen in order to control for the environmental aspect.

### 2.5. Procedure

The study has been approved by the Sheba IRB-Helsinki Committee (No. 4069-17-SMC) and by the Ethics Committee at Tel Aviv University (No. 0000275-2). Informed consent was obtained from all participants. This trial was registered on 31 October 2017 (https://clinicaltrials.gov/ct2/show/NCT03510377) (accessed on 26 October 2021) and conformed to the CONSORT checklist. All experiments were performed in accordance with relevant guidelines and regulations.

The participants of the study were recruited from older adults’ care centers and through social media (Facebook). All participants were independent and consented to the study by signing a consent form provided by a research assistant. 

Enrollment began in April 2018. All participants who met the inclusion criteria for the pilot study were allocated to three intervention groups: 6 participants attended the structured Ai-Chi, 7 participants the structured on-land Ai-Chi, and 6 participants the guided imagery of Ai-Chi. Simple randomization was generated by a research coordinator. 

Five people from the guided imagery of the Ai-Chi group dropped out of the study. Therefore, five additional people were recruited only for this group. As a result, 26.31% of the participants were not randomly allocated.

Testing was conducted at baseline and after 12 weeks of intervention by a qualified occupational therapist and qualified learning disability teacher. The brain scan was performed by a qualified technician while one of the evaluators conducted the cognitive test during the scan. The data was analysed by a qualified neuroscientist and a neuro-radiologist. The qualified technician and the neuroscientist and a neuro-radiologist did not know the arm of treatment (it was a blinded assessment). 

### 2.6. Statistical Analysis

All analyses were performed using IBM-SPSS v.23 (IBM-SPSS Statistics for Windows, IBM Corp., Armonk, NY, USA). A two-sided *p*-value ≤ 0.05 is considered statistically significant. A chi-square test of goodness-of-fit was performed to determine the goodness of fit of bio-demographic parameters between the three intervention groups. Since the dependent variables had a normal distribution, a One-way ANOVA analysis was conducted to ascertain if the participants did not differ in the experimental parameters measured between groups at the beginning of the intervention. A paired t-test was used to determine within-subject changes between pre-intervention (time 1) to post-intervention (time 2) values for each intervention group. A mixed design analysis of variance (ANOVA) was used to test the difference between intervention groups on performance in balance, 2-back score and brain activation over a period of 12 weeks, with time (Time 1, Time 2) as a within-subject factor and group (Ai-Chi, On-land Ai-Chi, Guided imagery of Ai-Chi) as a between-subjects factor.

f-MRI analysis: f-MRI data was processed using SPM12 [25], implemented in MATLAB. The pre-processing of functional images included reorienting, realignment to the first image using affine transformation, co-registration to the participant’s 3D T1 images, normalization to MNI stereotactic space, and smoothing with a Gaussian kernel of 8 × 8 × 8 mm^3^ to minimize anatomical differences and increase the signal-to-noise ratio. Following pre-processing, images were analyzed for each participant using the general linear model to produce two contrasts of interest: low working memory load: 1-back minus 0-back and high working memory load: 2-back minus 0-back. These contrasting images were then entered into a second-level whole-brain random effect analysis, comparing post- and pre-intervention scans. Study hypotheses were tested on these two contrasts of interest using a paired t-test (uncorrected, *p* < 0.001, k > 10). Due to the a priori interest of this study in the cerebellar activation, additional region of interest analyses were conducted using a cerebellar mask identified with the ‘Human Automated Anatomical Labelling (AAL) atlas’ [26] within the Wake Forest University PickAtlas and extracted using the MarsBaR ROI toolbox [27] as implemented in SPM8.

## 3. Results

There were no statistical differences between intervention groups in all bio-demographic variables and all measured parameters at baseline (Table 1). Means and standard deviations of 2-back scores and Tinetti balance test are presented in Table 2.

Paired T-test analyses revealed that only the Ai-Chi intervention group presented a significant change in balance between pre and post intervention (t = −4.8, *p* < 0.01). The on land Ai-Chi and the guided imagery of Ai-Chi intervention groups did not show significant differences between the two time points (on land Ai-Chi: t = −1.33, *p* > 0.05; guided imagery of Ai-Chi: t = −1, *p* > 0.05). 

A significant interaction between time (Time 1 and Time 2) and group (Ai-Chi, on-land Ai-Chi, guided imagery of Ai-Chi) was observed (F(2, 16) = 3.74, *p* < 0.05), demonstrating significant differences in balance abilities between intervention groups following intervention. Bonferroni post-hoc analyses indicated that the Ai-Chi group achieved a better improvement in the balance score over time than the on-land Ai-Chi and guided imagery of Ai-Chi groups. The on-land Ai-Chi group and the guided imagery of Ai-Chi group did not differ significantly from each other.

No significant differences were found between the intervention groups in 2-back scores between pre and post intervention (Ai-Chi: t = −1.73, *p* > 0.05; on land Ai-Chi: t = −0.93, *p* > 0.05; guided imagery of Ai-Chi: t = 1.19, *p* > 0.05).

No significant interaction between time (Time 1 and Time 2) and group (Ai-Chi, on-land Ai-Chi, guided imagery of Ai-Chi) was observed (F(2, 16) = 1.5, *p* > 0.05), demonstrating no significant differences in 2-back scores between intervention groups following intervention.

**Figure 2:**
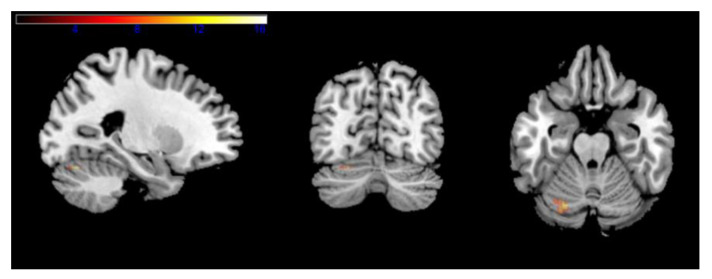
Effect of Ai-Chi on the high working memory load brain activation in an n-back task. Maximum intensity projections in three planes of the brain, depicting areas of significant activation (uncorrected, *p* < 0.001, k > 10) in a one-tailed-t statistic contrasting MR signal. The color scale shows t-values to the right.

In the whole-brain analysis, no significant differences in activation were found in the low working memory load. Nonetheless, in the high working memory load, the Ai-Chi group presented a decrease in cerebellar activation located in the left cerebellum_6 (based on the AAL atlas) (Figure 2) (Table 3).

Region of interest analyses yielded similar results by which pre-cerebellar activation was higher than post-intervention (M(SD) = 0.42(0.51), M(SD) = 0.19(0.41); t = 2.77, *p* < 0.05). The on-land Ai-Chi and the guided imagery of Ai-Chi intervention groups did not show any significant differences in activation between the two time points (on land Ai-Chi t = 1.04, *p* < 0.05; guided imagery of Ai-Chi t = −0.94, *p* < 0.05).

## 4. Discussion

The first aim of this pilot study was to examine the effects of Ai-Chi intervention for older adults on balance compared to identical intervention on-land and identical cognitive non-physical intervention. Our results showed significant differences in balance abilities between intervention groups following intervention. We found that after 12 weeks of intervention, only the Ai-Chi intervention group significantly improved their balance abilities. These findings are in line with previous studies that found a positive effect for Ai-Chi intervention and balance [18,28,29]. By using the characteristic of water (such as buoyancy, viscosity, turbulence, and hydrostatic pressure) and its influence on the human body, water-based physical activity such as Ai-Chi, has been suggested to improve balance control [30].

The second aim of this pilot study was to examine the effects of Ai-Chi intervention for older adults on cerebral activation during a working memory task compared to identical on-land and cognitive non-physical interventions. Our results showed no significant differences in 2-back scores between intervention groups following intervention. In addition, the Ai-Chi group presented a decrease in cerebellar activation located in the left cerebellum_6 in the high working memory load (2-back task). Region of interest analyses yielded similar results by which pre-cerebellar activation was higher than post-intervention. However, the on-land Ai-Chi and the guided imagery of Ai-Chi intervention groups did not show any significant differences in activation between the two time points (pre-intervention and post-intervention). Ai-Chi is characterized as a sequence of continuous movements performed while standing in shoulder-deep water [17]. This provides a multisensory stimulation combining the vestibular, the proprioception and the tactile systems. Immersion increases the proprioceptive input and sensory feedback to the immersed body [31].

The cerebellum has been known to play an integral role in sensorimotor control [32]. The vestibular system is connected to the cerebrum via the brain stem and cerebellum. The tactile and proprioceptive system provide external feedback to the cerebellum, allowing for regulation of motor activity. Previous studies also found decreased activation in the cerebellar cortex during the acquisition of a sequence of finger movements [33,34]. These studies suggest the contribution of the cerebellar cortex declines as proficiency at performing the task improves. Since no differences were found between the intervention group in the performances of the working memory task, our results of decreased activation in the cerebellum may thus represent a motor learning process, which leads to a less widespread but more targeted activation. 

The current study provides a neurobiological reinforcement to the concept of the relationship between an aquatic environment, balance, and cognitive improvement for abilities such as high working memory load. To the best of our knowledge, this is a novel concept.

### Limitations

Given its small sample size and the fact that 26.31% of participants were not randomly allocated, caution is advised before generalizing the results. In addition, no correction for multiple comparisons throughout voxels have been performed. Future studies should consider a larger study population and should test long term effects.

## 5. Conclusions

In conclusion, the current study provides evidence that Ai-Chi can be useful in improving balance in older adults as compared with on-land Ai-Chi and guided imagery of Ai-Chi. In addition, Ai-Chi practice decrease cerebellar activation in the left cerebellum in high working memory load task.

This study has practical implications in several spheres. In the sphere of aging, with advancing age, many adults report difficulties in balance and cognitive abilities. These abilities are essential for older adults to perform instrumental activities of daily living (IADL) in order to remain independent within the community. Ai-Chi may serve as an available and non-invasive intervention method that may help to decrease aging effects.

This study findings may have influence on occupational therapists, physiotherapists, and physical activity instructors when they make decisions concerning therapeutic intervention for older adults. In the sphere of hydrotherapy, the research highlights the current lack studies in this sphere, as well studies on the expanded contribution of aquatic intervention to spheres of functioning that are important to everyday living. In the sphere of neuroscience, one of the most studied areas in neuroscience is aging. In the last few years, the role of the cerebellum in cognitive processing has become better recognized. Technological and methodological advances in neuroimaging humans, together with findings in behavioral capabilities, provide new insights into the neuronal mechanisms that underlie both motor and cognitive abilities. This study allows a better understanding of the extent to which the cerebellum may be a mediator in the relationship between balance ability, working memory function and Ai-Chi practice.

## Figures and Tables

**Table 1:** Participants’ demographic variables across intervention groups and experimental parameters measured between groups at the beginning.

	*n*	On-Land Ai-Chi	*n*	Ai-Chi	*n*	Guided Imagery of Ai-Chi	Sig.
	7		6		6		
Gender							n.s
Male		2 (28.6%)		3 (50%)		2 (33.3%)	
Female		5 (71.4%)		3 (50%)		4 (66.7%)	
Age (years)		74 (5.9)		73.3 (7.4)		77 (6.9)	n.s
Tinetti balance (0–16)		13.8 (1.4)		13.5 (1.8)		14.5 (0.5)	n.s
2-back correct answers (0–5)		8 (2.4)		9 (2)		9.6 (1.9)	n.s

**Table 2:** Means and standard deviations of all parameters.

		On-Land Ai-Chi		Ai-Chi		Guided Imagery of Ai-Chi	
		Time 1	Time 2	Time 1	Time 2	Time 1	Time 2
Tinetti balance	M	13.8	14.4	13.5	15	14.5	14.6
	SD	1.4	1.6	1.8	1.5	0.5	0.5
2-back (correct answers)	M	8	9	9	10	9.6	8.6
	SD	2.4	1.4	2	1.6	1.9	2

**Table 3:** N-back activation. Table presents regions of activation resulting from a paired t-test threshold to a value of 0.05, k > 10, uncorrected. x, y, z are MNI coordinates for maximum Z score of Peak-level; 2 > 0-back = high working memory load.

		Peak MNI Coordinates (mm)			Peak-Level		Label (AAL)
2 > 0-back	# voxels	X	Y	Z	T-score	Z-score	
	31	−24	−70	−22	16.33	4.32	Cerebellum 6
		−32	−66	−22	8.12	3.5	Cerebellum 6
		−18	−76	−20	7.54	3.41	Cerebellum 6

## Data Availability

The datasets used and/or analyzed during the current study are available from the corresponding author on reasonable request.

## Supplementary Materials

The following are available at https://www.mdpi.com/article/10.3390/ijerph182312756/s1. Table S1: Intervention protocol.
