# Supplementary material for: Effects of Ai-Chi Practice on Balance and Left Cerebellar Activation during High Working Memory Load Task in Older People: A Controlled Pilot Trial
Source: Int J Environ Res Public Health. 2021 Dec 3;18(23):12756. doi: 10.3390/ijerph182312756 (PMC8656797; doi:10.3390/ijerph182312756)
Supplement: Supplementary file 1 [file ijerph-18-12756-s001.zip › ijerph-1457674-supplementary.pdf]

Supplementary Table S1. Illustration * of the Intervention Protocol (16 Ai-Chi movements).

1. Contemplating (X5 times)	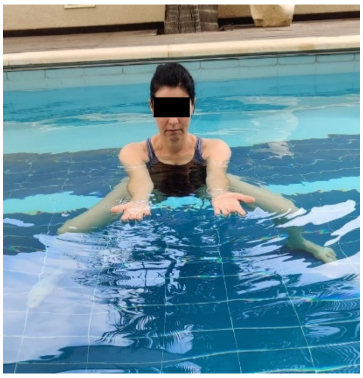 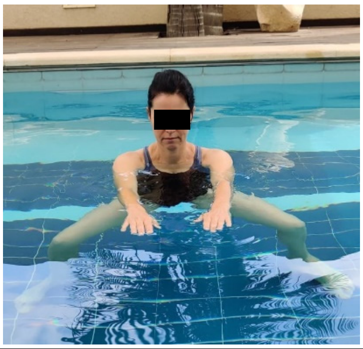
2. Floating (X5 times)	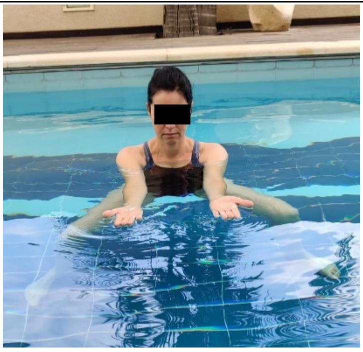 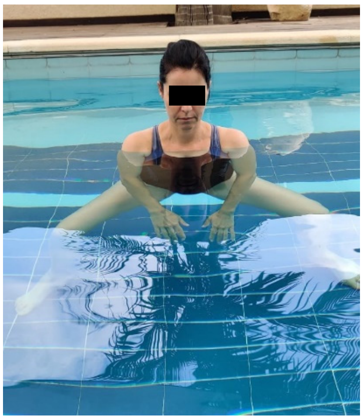

3. Uplifting<br>(X5 times)	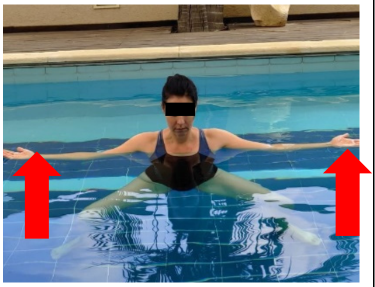<br>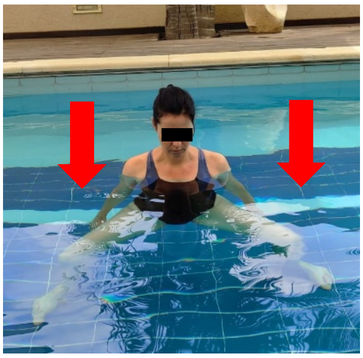
4. Enclosing<br>(X5 times)	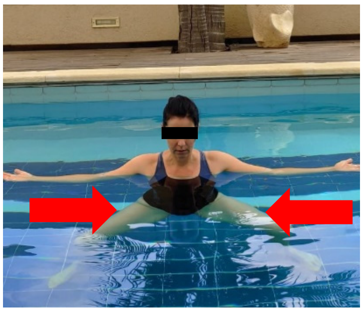<br>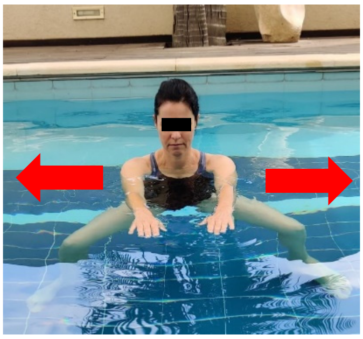

5. Folding<br>(X5 times)	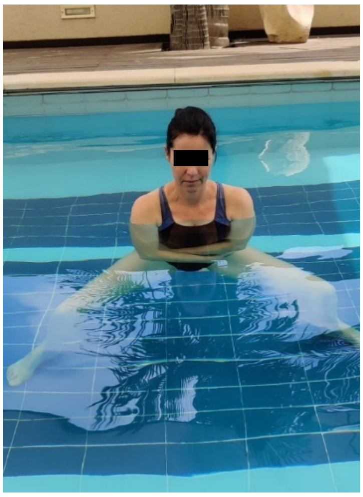<br>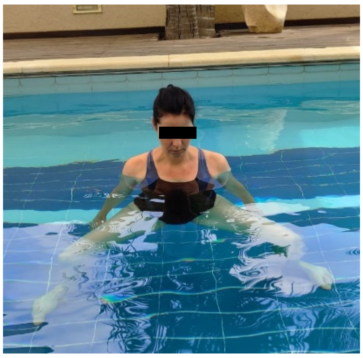
6. Soothing<br>(X3 times right &<br>X3 times left)	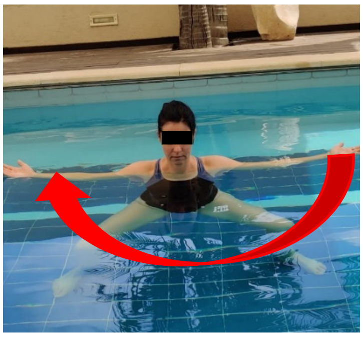<br>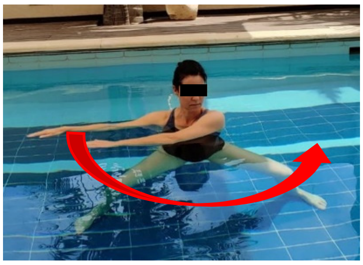

7. Gathering<br>(X3 times right &<br>X3 times left)	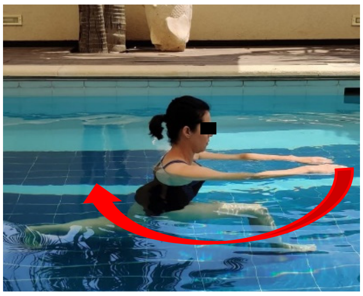<br>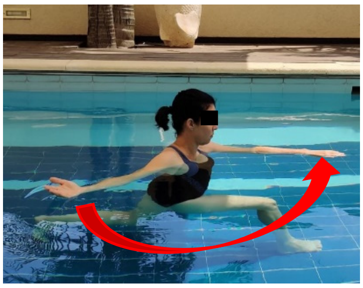
8. Freeing<br>(X3 times right &<br>X3 times left)	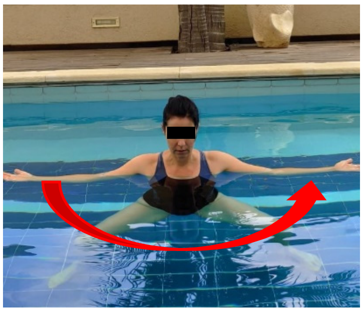<br>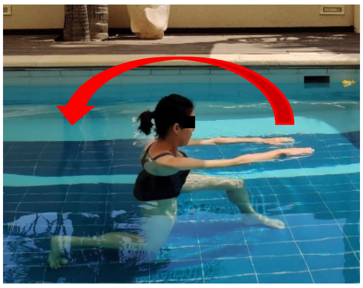<br>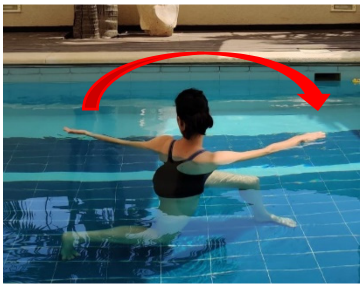

9. Shifting<br>(X3 times right &<br>X3 times left)	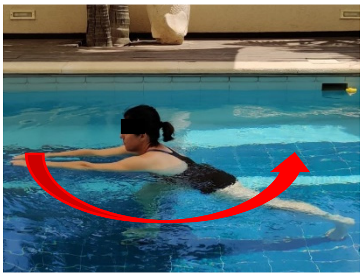<br>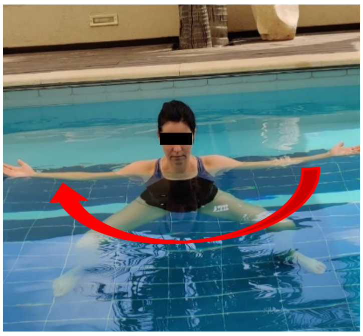
10. Accepting<br>(X3 times right &<br>X3 times left)	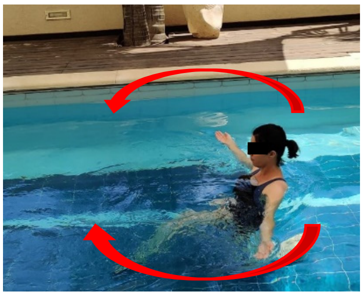<br>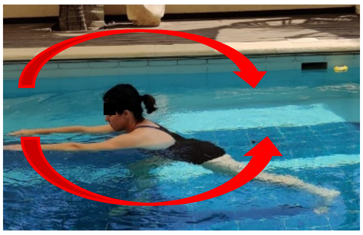
14. Encircling<br>(X3 times right & X3 times left)	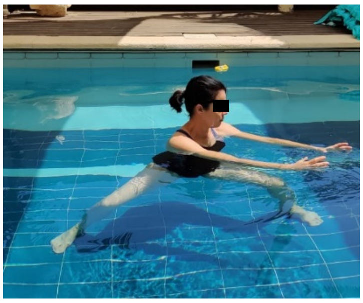

	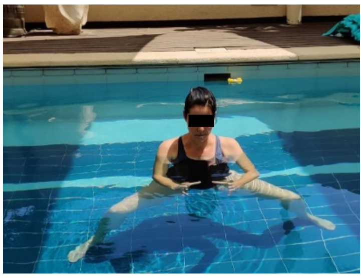
15. Surrounding<br>(X3 times right & X3 times left)	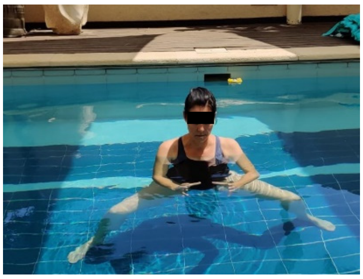<br>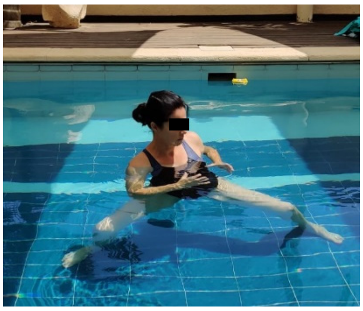
16. Nurturing<br>(X3 times right & X3 times left)	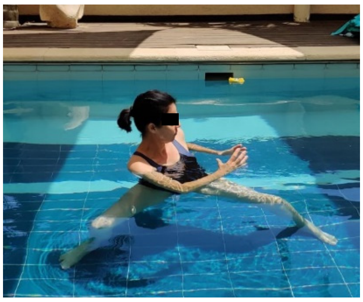

* The photos attached to this intervention protocol are an illustration of the intervention protocol and include a person that did not participate in this study. The person is a professional hydrotherapist who agreed to present the movements of the intervention protocol specifically for the purpose of this manuscript.
